# First cytogenetic characterization of a species of the arboreal ant genus *Azteca* Forel, 1978 (Dolichoderinae, Formicidae)

**DOI:** 10.3897/CompCytogen.v6i2.2397

**Published:** 2012-03-16

**Authors:** Danon Clemes Cardoso, Maykon Passos Cristiano, Luísa Antônia Campos Barros, Denilce Meneses Lopes, Silvia das Graças Pompolo

**Affiliations:** 1 Programa de Pós-graduação em Genética e Melhoramento, edifício Arthur Bernardes, subsolo sala 12, Universidade Federal de Viçosa, Viçosa, Minas Gerais, 36570-000, Brazil; 2 Departamento de Biologia Geral, Av. Peter Henry Rolfs s/n, Universidade Federal de Viçosa, Viçosa, Minas Gerais, 36570-000, Brazil

**Keywords:** karyotype, chromosome number, chromosome banding, ants, *Azteca trigona*

## Abstract

In this paper we present, for the first time, a detailed karyotype characterization of a species of the genus *Azteca* (Dolichoderinae, Formicidae). Cerebral ganglia from *Azteca trigona* Emery, 1893 were excised and submitted to colchicine hypotonic solution and chromosomal preparations were analyzed through conventional staining with Giemsa, C-banding, silver nitrate staining (AgNO_3_) and sequential base-specific fluorochromes. The analysis shows that *Azteca trigona* has a diploid number of 28 chromosomes. The karyotype consists of five metacentric pairs, seven acrocentric pairs and two pseudo-acrocentric pairs, which represents a karyotype formula 2K= 10M + 14A + 4A^M^ and a diploid number of the arms 2AN = 38. The analysis of heterochromatin distribution revealed a positive block on distal region of the short arm of fourth metacentric pair, which was coincident with Ag-NOR band and CMA_3_ fluorochrome staining, meaning that rDNA sequences are interspaced by GC-rich base pairs sequences. The C-banding also marked short arms of other chromosomes, indicating centric fissions followed by heterochromatin growth. The karyotype analysis of *Azteca trigona* allowed the identification of cytogenetic markers that will be helpful in a difficult taxonomic group as *Azteca* and discussion about evolutionary aspects of the genome organization.

## Introduction

The subfamily Dolichoderinae presents a great diversity of species throughout the world. Species are distributed in different biogeographic regions, from the Palearctic, Nearctic, Afrotropical region and Malaysia, to the Middle East, Australian and Neotropical regions (Bolton 1994). This subfamily comprises 22 genera, of which ten are found in Brazil, and *Azteca* Forel, 1878 and *Dolichoderus* Lund, 1831 form the two most diverse genera (Cuezzo 2003). The species of this subfamily represent a common group, but rather inconspicuous of Neotropical ant fauna compared to other more evident groups (Wild 2009, Cardoso and Cristiano 2010). One of the best known species of Dolichoderinae is the ant *Linepithema humile* (Mayr, 1868). This is one of the principal invasive ant species and now occurs in more than 15 countries (Diehl-Fleig and Diehl 2007, Wild 2009).

The genus *Azteca* is strictly Neotropical and very diverse, including around 130 species. They are essentially arboreal and many species have mutualistic associations with particular plant species, where the genus *Cecropia* presents the most conspicuous association (Longino 1991). Taxonomic reviews of the genus are scarce, regional or restricted to specific groups (Longino 2007) and the absence of a more comprehensive taxonomic review of the genus is the main obstacle for understanding the evolutionary basis of the *Azteca-Cecropia* mutualistic relationship.

Cytogenetic characterization offers some of the most reliable taxonomic criteria for some groups of organisms and recently, the application of cytogenetic studies focused on understanding the distribution pattern and evolution of species seems very promising (Mariano et al. 2012). In general, the parameters used in these studies are the number of chromosomes, their morphology, amount of heterochromatin, as well as their composition and the base pairs, obtained by chromosome banding techniques. Extensive information on chromosomes of the order Hymenoptera is available, especially the Formicidae of which there are already many known karyotypes. However cytogenetic information is lacking for a great part of known species. At least 750 from more than 12000 species have been cytogenetically studied (Lorite and Palomeque 2010), and the variation in chromosome number is enormous, ranging from 2n=2 to 2n=120 (Mariano et al. 2008). This variation can provide trustworthy cytotaxonomic markers for evolutionary studies in association with chromosome banding. At least 16 genera of the subfamily Dolichoderinae have their chromosome numbers available (Lorite and Palomeque 2010), where the genus *Iridomyrmex* Mayr, 1862 is the most studied (including the species *Iridomyrmex humile*, now relocated to the genus *Linepithema* Mayr, 1866 (Wild 2009)). The chromosome number in Dolichoderinae is less variable than in other related subfamilies, ranging from 2n= 10 to 2n= 48 (Lorite and Palomeque 2010). Within genera, the chromosome number varies from 2n=14 to 48 in *Iridomyrmex* and represents the largest variation found in the subfamily, while, *Dolichoderus* showed karyotypes ranging from 2n=18 to 30 (Imai et al. 1984a, b).

Even with the immense diversity of species of the genus *Azteca*, including approximately 130 described species, no cytogenetic study is encountered for this genus. Thus, to contribute to the increased cytogenetic knowledge of Formicidae and further understanding of karyotype evolution, the present study aimed to characterize the karyotype of *Azteca trigona* Emery, 1893, whereas karyotypes of the genera *Anillidris* Santschi, 1936 and *Liometopum* Mayr, 1861 remain totally unknown.

## Material and methods

Thirty specimens from two colonies of *Azteca trigona* collected in Ponte Nova (20°25'S, 42°54'W) and Viçosa (20°45'S, 42°52'W), MG, Brazil were analyzed. The colonies were collected in the field and transferred to a plastic container and maintained in a BOD (Biochemical Oxygen Demand) incubator at 25°C following the protocol described by Cardoso et al. (2011) and fed with honey in order to obtain larvae in the pre-pupa stage (post-defecating larvae). The specimens were identified by specialists and Vouchers of the samples collected in this work were deposited in CEPLAC and MZUSP.

Cytogenetic analysis was performed using cerebral ganglia of the larvae selected. Metaphase chromosomes were obtained according to the methodology proposed by Imai et al. (1988). Preparations obtained from fifteen individuals per colony were analyzed. The preparations were stained with Giemsa diluted in Sörensen buffer at (4%) for 20 minutes. On average, ten metaphases were analyzed per slide and ten slides were submitted to banding techniques. C-banding was performed by BSG method (Barium hydroxide/Saline/Giemsa) according to Sumner (1972). The protocol of Schweizer (1980) was used for preparation of sequential fluorochrome staining (CMA_3_/DA/DAPI). Identification of nucleolus organizer regions (NOR) was performed according to Howell and Black (1980). The best metaphases were photographed using an Olympus BX60 microscope equipped with a camera Q color 3 Olympus®. Brightness and contrast of the karyotypes were optimized using Photoshop CS4. The karyotypes were mounted in Corel Draw® 13 image editing software. Karyotype structure was described according to the nomenclature proposed by Imai (1991) and Levan et al. (1964). For mounting of the karyotypes, the chromosomes were sorted into three groups: metacentric chromosomes (M), acrocentric chromosomes (A) with heterochromatin located across the length of the short arm of the chromosomes, and pseudo-acrocentric chromosomes (A^M^) which possess a long heterochromatic arm (Imai 1991).

## Results and discussion

**Figure 1.**
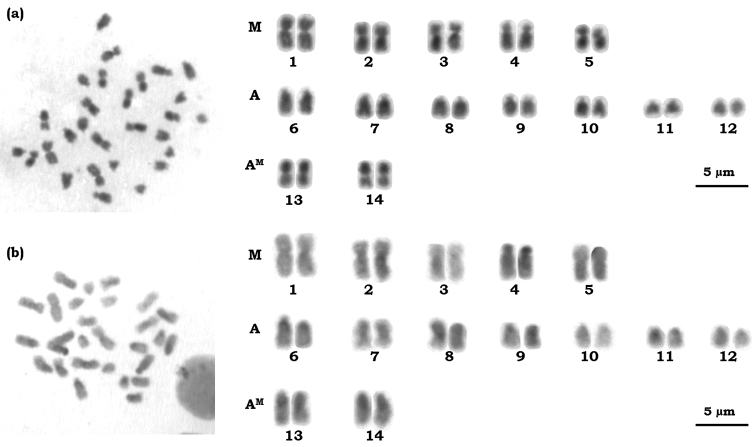
Karyotype of female workers of *Azteca trigona* 2n = 28 sorted according to the classification proposed by Imai (1991) **a** Conventional staining using Giemsa **b** C-banding showing the distribution of heterochromatin.

The chromosome number observed for *Azteca trigona* was 2n = 28 (Figure 1). The karyotype of this species consists of five metacentric pairs (M), seven acrocentric pairs and two pseudo-acrocentric pairs (A^M^) according to the terminology proposed by Imai (1991). Considering this chromosome classification, the karyotype formula found for the diploid set would be 2K= 10M + 14A + 4A^M^. Thus, the diploid number of the arms according to Imai et al. (1994) was 2AN = 38. However, considering Levan et al. (1964), the arm ratio analysis results in five pairs of metacentric (pairs 1, 2, 3, 13 and 14), two pairs of submetacentric (pairs 4 and 5) and 7 pairs of subtelocentric (pairs 6 to 12). It is important to emphasize that the chromosome classification by Levan et al. (1964) is the most used in cytogenetic studies of Formicidae (Lorite and Palomeque 2010), although classification proposed by Imai (1991) is more informative from an evolutionary view. It was not possible to observe the haploid karyotype (n) since there was no production of alates (reproductive individuals) during the maintenance period of the colony.

Of the subfamilies of Formicidae, Dolichoderinae is the fourth subfamily with the major number of taxa studied. According to Lorite and Palomeque (2010), about 50 taxa in 16 genera have been studied. The diploid karyotype of Dolichoderinae varied from 10 chromosomes in *Tapinoma indicum* Forel, 1895 and *Tapinoma melanocephalum* (Fabricius, 1793) (Imai et al. 1984a), up to 48 chromosomes in *Iridomyrmex anceps* (Roger, 1863) (Imai et al. 1984b). However, the latter chromosome number has been questioned due to the great discrepancy from other karyotypes described to subfamily (Lorite and Palomeque 2010). The chromosome number of *Azteca*,2n = 28, seems consistent with the karyotypic variation of the subfamily, which according to Crozier (1970) and Imai et al. (1977) is characterized by a low to average chromosome number.

**Figure 2.**
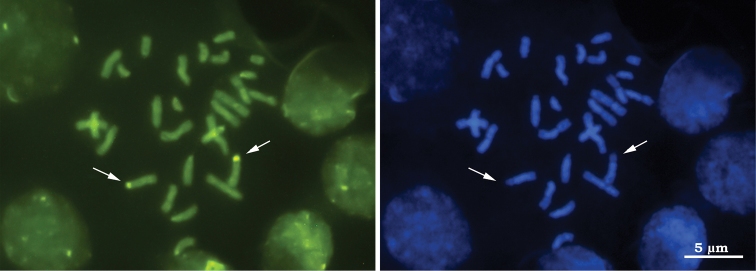
Diploid metaphase of *Azteca trigona* submitted to sequential staining with fluorochromes CMA_3_/DA/DAPI **a** Staining with CMA_3_, arrows indicate the fourth chromosome pair and the GC^+^ regions **b** Staining with DAPI, arrows indicate the same chromosome pair with the negative AT-rich regions.

**Figure 3.**
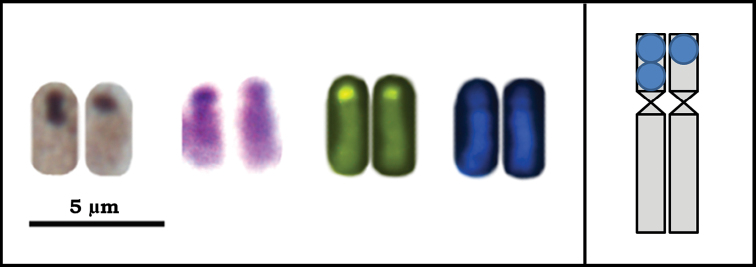
Fourth pair of chromosomes of *Azteca trigona* submitted to different banding techniques: Ag-NOR, C-banding and sequential CMA_3_/DA/DAPI fluorochrome staining. The inserted scheme indicates that for all techniques the homologues are heteromorphic for the banding pattern.

Results of the banding techniques indicate positive C-, Ag-NOR and CMA_3_-bands, and negative DAPI-bands on the short arm of the fourth pair of metacentric chromosomes (Figs 1–3), indicating that this region is rich in heterochromatin and should correspond to the nucleolus organizer region (NOR) (Fig. 3). This chromosome pair was CMA_3_-positive and DAPI-negative, indicating that the marked regions are rich in GC bases and devoid of AT bases (Fig. 2). Several authors have reported that CG-positive and AT-negative (i.e. auto-complementary) regions are related to nucleolus organizer regions (NORs). This relationship has been reported in grasshoppers (Camacho et al. 1991), bees of the genus *Melipona* (Illiger, 1806) (Rocha et al. 2002) as well as in other neotropical bees (Rocha et al. 2003; Brito et al. 2003) and in ants *Dinoponera lucida* Emery, 1901 (Mariano et al. 2008) and *Tapinoma nigerrimum* (Nylander, 1856) (Lorite et al. 1997), where the latter belongs to the same subfamily of ants as *Azteca trigona*. In addition, in many studies homology between the Ag-NOR positive regions and probes specific to NORs was revealed by means of the *in situ* hybridization technique (Lorite et al. 1997, Rocha et al. 2002, Mariano et al. 2008). Since this technique uses specific probes, it is much more sensitive to detection of NORs and provides consistent data on the relationships between banding patterns – C-bands, Ag-NOR and fluorochrome staining.

Furthermore, the silver nitrate staining revealed that the fourth metacentric pair also had an Ag-positive block. This finding corroborated that this pair carried NORs, which were heteromorphic between the homologues (Fig. 3). Heteromorphism in NOR size is frequent in a large number of organisms and can be explained by tandem duplications of the ribosomal genes (Sumner 2003). The NOR heteromorphism found in *Azteca trigona* probably resulted from the duplication/amplification or unequal crossover during meiosis of some ribosomal sequences of the homologues.

The results presented here are, to our knowledge, the first cytogenetic data of a species of the genus *Azteca*, and the second known for a species of the subfamily Dolichoderinae in the Neotropics. Previously, cytogenetic data on the species *Dorymyrmex pyramicus* (Roger, 1863) (2n = 18) were presented on the base of only five workers collected in Uruguay (Gõni et al. 1983). According to some authors, cytogenetic data on Neotropical ant species are scarce given the immense biodiversity of this region (Goñi et al. 1983, Lorite et al. 1997). Cytogenetic data are important tools that can be used for phylogenetic inferences (Rocha et al. 2002, Lorite and Palomeque 2010) and solving species identification problems (Borges et al. 2004, Delabie et al. 2008).

In particular, the genus *Azteca* presents a great challenge to taxonomists since identification is practically impossible at the species level in absence of the queen (Longino 2007). Since it is hardly possible to identify the species only by workers, it becomes a limiting factor for studies on biology, ecology and biodiversity of the genus, because the workers they are much more abundant in the colony and the queen is not always collected. Cytogenetic data presented herein are the first records for the genus and can be used for the development of cytogenetic markers. These may be used in phylogenetic inference and applied to taxonomy in order to facilitate the identification of species. Increase in the number of samples and application of other banding techniques to acquire cytogenetic patterns typical of the species of the genus are to be conducted for the better understanding of evolution and taxonomy of this group.
